# Social support and self-efficacy mediate the link between health literacy and fear of disease progression in Chinese lung cancer patients

**DOI:** 10.1038/s41598-026-56265-3

**Published:** 2026-06-29

**Authors:** Ling Zhang, Jing Deng, ZhiFang Li, Fanghui Wu, Ting Luo, Fengju Li, Yumei Shi, Chuanfen Zheng, Honghui Rong, Lu Lu, Enyu Lei, Ji-an Chen

**Affiliations:** 1 Department of Health Education, College of Military Preventive Medicine, Army Medical University, Chongqing, 400038 China; 2 Gastric Cancer Center, West China Hospital, Sichuan University, Sichuan, 610000 China; 3 West China School of Nursing, Sichuan University, Sichuan, 610000 China; 4 Department of Foreign Languages, College of Basic Medical Sciences, Army Medical University, Chongqing, 400038 China; 5 Department of Basic Psychology, School of Psychology, Army Medical University, Chongqing, 400038 China; 6 Affiliated Cancer Hospital of Chongqing University, Chongqing, 400038 China

**Keywords:** Fear of disease progression, Health literacy, Self-efficacy, Social support, Cancer, Health care, Medical research, Oncology, Psychology, Psychology

## Abstract

Fear of disease progression (FOP) has emerged as one of the most prevalent and distressing unmet needs among lung cancer patients in recent years. This study aimed to explore the associations between health literacy (HL), social support (SS), self-efficacy (SE) and FOP, and to examine the mediating roles of SS and SE in the relationship between HL and FOP in lung cancer patient population. A cross-sectional design with convenience sampling was employed to survey cancer inpatients in two general hospitals in Chongqing and Chengdu, two major cities in southwestern China. Data were collected via validated scales for HL, SE, SS and FOP. A structural equation model (SEM) was then constructed, *P* ≤ 0.05 was considered statistically significant. The mean FOP score FOP was 31.20 ± 9.28; 162 patients (43.09%) exceeded cutoff for psychological dysfunction (≥ 34). Correlation analysis revealed that FOP was significantly negative correlated with all variables except for objective support (OS) and subjective support (Subs) (*p* < 0.01). The SEM of the FOP exhibited good fit (GFI = 0.949, NFI = 0.964, CFI = 0.978, IFI = 0.978, AGFI = 0.918, RMSEA = 0.064). The model indicated that HL had the strongest correlation with FOP (β = -0.300, *p* < 0.05), followed by SE (β =−0.281, *p* < 0.05). Notably, SE mediated the association between HL and FOP (contribution effect: 40.67%). SS positively influence SE (β = 0.131, *p* < 0.05) but had no statistically significant effect on FOP (*p* > 0.05). HL exerted the strongest protective effect on FOP, both directly and indirectly via enhanced SE. Utilization of support Subs (US) was clinically more significant than OS and Subs. Clinical interventions should prioritize improving health literacy through targeted education, psychological support, and coping skills training to strengthen patients’ use of available support and foster psychological adaptation.

## Introduction

Lung cancer remains the leading cause of cancer incidence and mortality worldwide, posing a severe threat to global public health. According to the Global Cancer Burden report released by the International Agency for Research on Cancer (IARC) in 2024, there were nearly 20 million new cancer cases globally in 2022, of which 2.48 million (12.4%) were lung cancer, making it the most commonly diagnosed malignancy. Among the 9.7 million cancer-related deaths reported worldwide, lung cancer accounted for 1.8 million deaths (18.7%), ranking as the foremost cause of cancer mortality^1,2^. The disease burden of lung cancer in China is particularly pronounced. In 2022, the country reported 1.061 million new lung cancer cases, 22.0% of its 4.825 million incident cancers, and accounted for 42.8% of global lung-cancer incidence, the highest national incidence globally^3^. Advances in medical technologies, equipment, and targeted therapies have markedly prolonged survival and improved quality of life among cancer patients^4^. Nevertheless, the persistent risk of recurrence and metastasis, together with the complexity of treatment regimens, engenders ongoing fear, worry, or concern regarding cancer recurrence or progression, collectively termed fear of progression (FOP)^5–7^. The prevalence of FOP among cancer patients ranges from 33% to 97%^8,9^. It has been recognized as one of the most frequent and distressing psychological challenges faced by cancer survivors. Elevated level of FOP not only impairs quality of life but may also undermines treatment adherence, drives excessive healthcare utilization, and predisposes to comorbid anxiety and depression^6^. Consequently, identifying key factors influencing FOP in lung cancer patients has become a critical focus in clinical and psychological intervention research.

Although FOP is highly prevalent and exerts a substantial impact on lung cancer patients, the underlying psychosocial mechanisms remain to be fully elucidated. In recent years, research has increasingly focused on individual-level psychosocial resources as key factors in modulating cancer-related distress, especially for social support (SS)^10,11^. Moreover, some studies have begun to explore the associations between health literacy (HL) or self-efficacy (SE) and FOP, but robust evidence is still lacking and warrants further in-depth investigation^12–15^.

HL refers to an individual’s ability to access, understand, and apply health information to make informed health decisions^16^. Evidence suggests that higher HL enables patients to better comprehend their condition and treatment plans, enhances perceived controllability of the disease, and thereby reduces FOP^11,13^. SS, especially emotional and practical support from family members, buffers psychological stress, fosters feelings of security and belonging, and thereby alleviate fears of recurrence or disease worsening^10,11^. SE defines as individual’s confidence in managing disease-related challenges. Patients with high SE are more likely to adopt active coping strategies, demonstrate greater treatment adherence, and exhibit improved emotional regulation, all of which contribute to lower levels of FOP^15^.

However, despite of preliminary evidence on the individual associations between HL, SS, SE, and FOP, few studies take into account the combined influence of FOP from multiple perspectives^17^, especially the relationship between HL and the other three constructs. The mechanisms through which these factors interact and jointly influence FOP in lung cancer patients remain unclear. Most current studies rely on simple correlation analyses or linear regression models, which are insufficient to capture the complex interrelationships among variables, particularly potential mediating or indirect effects.

According to Social Cognitive Theory^18^, individuals’ self-efficacy is closely linked to their ability to comprehend and manage task-relevant information. Patients with higher HL are more capable of interpreting medical information and acquiring symptom management skills, which in turn enhances their SE in managing illness. Conversely, limited basic health knowledge and poor information-processing abilities may lead to feelings of helplessness and uncertainty, thereby diminishing patients’ sense of behavioral control.

HL also affects the recognition, seeking, and effective utilization of SS. Although SS may be objectively accessible, individuals’ ability to actively identify avaible resources, appropriately express their needs, and effectively integrate external assistance depends heavily on their health communication and health decision-making competencies─core dimensions of health literacy^19^.

Therefore, to deepen the understanding of the psychosocial mechanisms underlying FOP, we propose a mediation model grounded in Social Cognitive Theory^18^ and the Integrated Health Literacy Framework^20^. The model posits that health literacy, as the foundational competence for processing health information, not only directly reduce FOP, but also indirectly buffers its impact by enhancing self-efficacy and optimising the use of social support. We tested this pathway in a cross-sectional survey of Chinese lung-cancer patients, measuring levels of FOP, HL, SS, and SE. Structural equation modeling (SEM) was employed to quantify both direct and indirect effects. The findings are intended to establish a theoretical foundation that can assist healthcare professionals in developing more precise and effective psychological interventions.

## Methods

### Study design and participants

In this cross-sectional survey study (January–March 2022), 400 in-patients with pathologically confirmed lung cancer were consecutively recruited from two tertiary hospitals in Chongqing and Chengdu, southwestern China. Convenience sampling was used to achieve the target sample size (*n* = 400) required for SEM (≥ 10 participants per free parameters, allowing for 5% invalid or incomplete questionnaires). Inclusion criteria were: (1) aged ≥ 18 years; (2) pathologically confirmed lung cancer; (3) able to provide written informed consent; (4) no cognitive impairment or major psychiatric disorder. Exclusion criteria were: (1) severe organ failure; (2) concurrent malignancies; and (3) comorbid psychiatric or cognitive conditions that would preclude valid participation. Of the 400 patients approached, 24 individuals discontinued because of questionnaire length or personal reasons, leaving 376 participants for analysis. Data were collected by trained oncology nursing staff who conducted the field investigation under standardized conditions. Questionnaires were administered face-to-face or self-completed, according to participant’s literacy and preference, to ensure response accuracy and reliability. The study was approved by the Ethics Committee of Chongqing University Cancer Hospital (Approval No. CZLS2022210-A).

### Instruments

#### General information questionnaire

The research team developed the self-report questionnaire based on a literature review and the study objectives. The general information included sex, age, marital status, occupation, monthly income, medical burden, place of residence, cancer stage, and other relevant demographics.

### Fear of progression

FOP was measured with the simplified Chinese version of the Fear of Progression Questionnaire-Short Form (FOP-Q-SF), adapted and validated by Wu et al.^21^ from the original scale of Mehnert et al.^22^. The 12-item instrument uses a 5-point Likert scale; total scores range from 12 to 60, with higher values indicating greater fear. A score ≥ 34 denotes clinically significant psychological distress and reflects a high level of FOP^23,24^. The FOP-Q-SF measures two subscales: physical health (PH, fear of declining health) and social family (SF, fear of impaired family functioning). Cronbach’s α in the present sample was 0.950.

### Health literacy

HL was evaluated with the Health Literacy Management Scale (HeLMS) for patients with chronic disease, originally developed by Jordan et al.^25^. and validated in Chinese by Sun et al.^26^. The scale comprises 24 items rated on a 5-point Likert scale (1–5), yielding a total score range of 24–120, with higher scores denoting better HL. Four subscales are extracted: (1) information acquisition ability (IAA, 10 items), (2) communication and interaction ability (CIA, 8 items), (3) health improvement willingness (HIW, 4 items), and (4) economic support willingness (ESW, 2 items). The Cronbach’s α coefficient was 0.980.

### Social support rating scale

SS was measured with the Social Support Rating Scale (SSRS), developed by Xiao et al. (1986)^27^ and widely used in Chinese populations^17,28^. The SSRS is a 10-item self-report instrument that assesses SS across three dimensions: objective support (OS, 4 items), subjective support (Subs, 3 items), and utilization of support (US, 3 items). The total score ranges from 12 to 66, with higher scores indicating greater SS. The scale demonstrates strong psychometric properties (test–retest *r* = 0.92; item consistency 0.89–0.91). Cronbach’s α coefficient was 0.824.

### Self-efficacy

SE was evaluated with the Chinese version of the Strategies Used by People to Promote Health (SUPPH) scale, originally developed by Lev and Owen^29^ and subsequently translated and validated by Qian Yun-hui et al.^30^. The scale consists of 28 items comprising three subscales: (1) positive attitude (PA, 15 items), (2) self-decision (SD, 3 items), and (3) self-decompression (SDP, 10 items)^31^. Each item is rated on a 5-point Likert scale, total and subscale scores are summed, with higher scores indicating greater confidence in managing health-related challenges. The SUPPH has demonstrated good reliability and validity in assessing SE among individuals with chronic conditions. Cronbach’s α coefficient was 0.981.

### Statistical analyses

Data were entered into a database EpiData version 3.02. To ensure accuracy, all questionnaires were coded and double-entered independently by two trained data managers. Descriptive statistics were used to summarize the data: categorical variables were presented as frequencies and percentages; continuous variables were reported as means and standard deviations (SD). Chi-square tests were conducted to compare the frequency distributions of categorical variables between the low and high FOP groups. Pearson correlation coefficients were calculated to examine the bivariate associations among study variables. SEM was performed using IBM SPSS AMOS 22.0 to test the hypothesized theoretical model (Fig). Maximum likelihood estimation with bootstrapping was applied to enhance parameter estimation robustness. Model fit was evaluated with multiple indices, including the comparative fit index (CFI), goodness-of-fit index (GFI), normed fit index (NFI), and root mean square error of approximation (RMSEA). The model was iteratively refined on both theoretical and statistical grounds to achieve optimal fit. A p-value < 0.05 was considered statistically significant for all analyses.

## Results

### Baseline characteristics

A total of 400 patients were screened, and 24(6.0%) were excluded, leaving 376 participants for analysis. The mean age was 56.48 ± 12.20 years; 241 (64.10%) were male; 361(96.01%) were Han nationality; 328 (87.23%) were married. 108 (28.72%) were at Stage II of lung cancer; and 323(85.90%) had the family history of cancer (Table 1).

**Table 1:** Sample demographics and the distribution of FOP across demographic characteristics(*n* = 376).

Characteristics	Categories	*N*(%)	FOP		χ^2^ Value	*P* Value
			Low	High		
Gender	Male	241(64.10)	135(56.02)	106(43.98)	0.221	0.638
	Female	135(35.90)	79(58.52)	56(41.48)		
Age	18~	67(17.82)	40(59.70)	27(40.30)	2.024	0.364
	45~	162(43.09)	97(59.88)	65(40.12)		
	60~	147(39.10)	77(52.38)	70(47.62)		
Ethnicity	Han	361(96.01)	204(56.51)	157(43.49)	0.606	0.436
	Other	15(3.99)	10(66.67)	5(33.33)		
Marital status	Unmarried/divorce/widowed	48(12.77)	24(50.00)	24(50.00)	1.073	0.300
	Married	328(87.23)	190(57.93)	138(42.07)		
Education level	Below primary school	112(29.79)	66(58.93)	46(41.07)	1.755	0.625
	Primary school	141(37.50)	83(58.87)	58(41.13)		
	High school	72(19.15)	40(55.56)	32(44.44)		
	University or higher	51(13.56)	25(49.02)	26(50.98)		
Residence	Rural	211(56.12)	127(60.19)	84(39.81)	2.103	0.147
	Urban	165(43.88)	87(52.73)	78(47.27)		
Annual household income	< 20,000 RMB	47(12.50)	25(53.19)	22(46.81)	4.286	0.232
	20,000 ~ 49,999 RMB	153(40.69)	80(52.29)	73(47.71)		
	50,000 ~ 99,999 RMB	123(32.71)	79(64.23)	44(35.77)		
	≥ 100,000 RMB	53(14.10)	30(56.60)	23(43.40)		
Current occupational status	Not employed	162(27.13)	97(59.88)	65(40.12)	3.191	0.203
	Retired	100(26.60)	60(60.00)	40(40.00)		
	Employed	114(46.28)	57(50.00)	57(50.00)		
Stage of Cancer	0-Ⅰ	92(24.47)	63(68.48)	29(31.52)	28.524	0.000
	Ⅱ	108(28.72)	71(65.74)	37(34.26)		
	Ⅲ	74(19.68)	34(45.95)	40(54.05)		
	Ⅳ	70(18.62)	24(34.29)	46(65.71)		
	Unable to judge or know	32(8.51)	22(68.75)	10(31.25)		
Family history of cancer	Yes	323(85.90)	190(58.82)	133(41.18)	3.404	0.065
	No	53(14.10)	24(45.28)	29(54.72)		

### Factors associated with FOP

The mean score of FOP was 31.20 ± 9.28 (range 12–58); 162 participants (43.09%) reached clinically dysfunctional threshold (≥ 34). Pearson’s chi-square (Table 1) revealed that higher FOP was significantly associated with cancer stage (*P* < 0.001).

### Descriptive statistics for measured variables

Table 2 summarizes the descriptive statistics (range, mean, standard deviation) and univariate skewness and kurtosis for the measurement variables. Skewness and kurtosis values lay within ± 2, indicating approximate univariate normality and justifying the use of parametric procedures. Mean scores were 96.34 ± 19.57 for HL, 41.06 ± 9.28 for SS and 85.55 ± 19.36 for SE.

**Table 2:** Descriptive statistics of the measured variables (*n* = 376).

Variables	Range	Dimension scores	Average score of entries	Skewness	Kurtosis
**HL**	**24 ~ 120**	96.34 ± 19.57	4.01 ± 0.82	–0.71	0.28
IAA	10 ~ 50	40.31 ± 8.38	4.48 ± 0.93	–0.61	–0.12
CIA	8 ~ 40	32.13 ± 6.80	3.57 ± 0.76	–0.73	0.25
HIW	4 ~ 40	16.30 ± 3.69	4.08 ± 0.92	–0.76	–0.11
ESW	1 ~ 10	7.60 ± 2.07	3.80 ± 1.04	–0.44	–0.60
**SS**	**18 ~ 59**	41.06 ± 9.28	4.11 ± 0.93	–0.50	–0.44
OS	8 ~ 33	24.20 ± 6.03	6.05 ± 1.51	–0.88	–0.04
Subs	2 ~ 19	8.79 ± 2.84	2.93 ± 0.95	0.42	0.54
US	3 ~ 12	8.08 ± 2.30	2.69 ± 0.77	0.22	–0.63
**SE**	**28 ~ 140**	85.55 ± 19.36	3.06 ± 0.69	0.20	0.89
PA	15 ~ 75	45.71 ± 10.56	3.05 ± 0.70	0.19	0.71
SD	3 ~ 15	9.18 ± 2.32	3.06 ± 0.77	0.22	0.26
SDP	10 ~ 50	30.65 ± 7.32	3.06 ± 0.73	0.28	0.65
**FOP**	**12 ~ 58**	31.20 ± 9.28	2.60 ± 0.77	0.08	–0.17
PH	6 ~ 30	15.71 ± 4.78	2.62 ± 0.80	0.13	0.04
SF	6 ~ 29	15.49 ± 4.89	2.58 ± 0.81	0.15	–0.24

### The correlations between HL, SE, SS and FOP

Table 3 presents the results of the correlation analyses of HL, SE, SS, and FOP. The Pearson correlation analyses showed that FOP was negatively significant correlated with the all variables except for OS and Subs (*p* < 0.01). HL, SE, and SS were positively inter-correlated (*p* < 0.01), although the correlations between Subs and both SD and SDP were not statistically significant.

### Measurement model

**Fig. 1:**
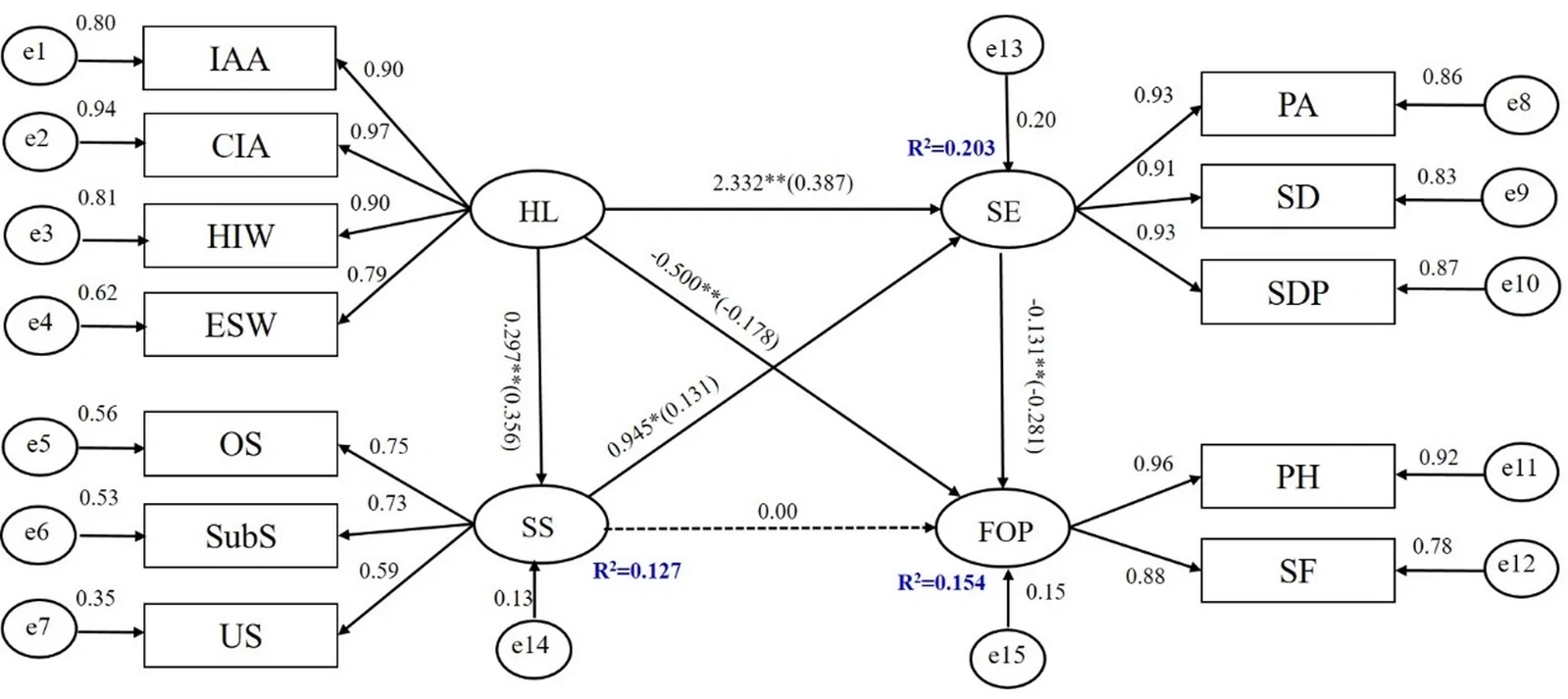
Standardized estimates of relationships and effect sizes in the structural model.

Because HL, SE, and SS were all associated with FOP, SEM was utilized to design a mediating model. According to the research hypothesis, a path analysis diagram of the whole model was constructed. However, the relationship between SS and FOP was not statistically significant. This non-significant path was therefore removed to improve model parsimony and fit. The final modified model (Fig. 1), demonstrates adequate goodness-of-fit indices: CFI = 0.978, GFI = 0.949, NFI = 0.964, IFI = 0.978, AGFI = 0.918, and RMSEA = 0.064. All observed variables showed significant and strong loadings on their respective latent constructs, with most standardized regression weights exceeding 0.70, confirming measurement reliability and convergent validity.

**Table 3:** Correlations (r) between HL, SE, SS and FOP.

variables	HL	IAA	CIA	HIW	ESW	SE	PA	SD	SDP	SS	OS	Subs	US	FOP	PH	SF
**HL**	1															
IAA	0.947^**^	1														
CIA	0.956^**^	0.854^**^	1													
HIW	0.886^**^	0.757^**^	0.851^**^	1												
ESW	0.821^**^	0.715^**^	0.751^**^	0.735^**^	1											
**SE**	0.384^**^	0.375^**^	0.342^**^	0.336^**^	0.398^**^	1										
PA	0.369^**^	0.354^**^	0.330^**^	0.333^**^	0.386^**^	0.963^**^	1									
SD	0.345^**^	0.338^**^	0.314^**^	0.297^**^	0.371^**^	0.873^**^	0.805^**^	1								
SDP	0.366^**^	0.354^**^	0.332^**^	0.324^**^	0.374^**^	0.939^**^	0.838^**^	0.833^**^	1							
**SS**	0.266^**^	0.241^**^	0.264^**^	0.225^**^	0.254^**^	0.223^**^	0.197^**^	0.226^**^	0.219^**^	1						
OS	0.176^**^	0.167^**^	0.162^**^	0.157^**^	0.177^**^	0.189^**^	0.160^**^	0.202^**^	0.180^**^	0.909^**^	1					
Subs	0.266^**^	0.235^**^	0.279^**^	0.235^**^	0.213^**^	0.103^*^	0.108^*^	0.095	0.094	0.778^**^	0.582^**^	1				
US	0.304^**^	0.276^**^	0.321^**^	0.228^**^	0.283^**^	0.268^**^	0.241^**^	0.252^**^	0.277^**^	0.666^**^	0.450^**^	0.411^**^	1			
**FOP**	–0.298^**^	–0.286^**^	–0.275^**^	–0.218^**^	–0.335^**^	–0.292^**^	–0.299^**^	–0.266^**^	–0.245^**^	–0.118^*^	–0.088	–0.025	–0.214^**^	1		
PH	–0.280^**^	–0.265^**^	–0.273^**^	–0.192^**^	–0.305^**^	–0.289^**^	–0.293^**^	–0.248^**^	–0.247^**^	–0.132^*^	–0.094	–0.037	–0.249^**^	0.953^**^	1	
SF	–0.304^**^	–0.293^**^	–0.272^**^	–0.243^**^	–0.337^**^	–0.273^**^	–0.286^**^	–0.253^**^	–0.222^**^	–0.100	–0.081	–0.019	-0.171^**^	0.961^**^	0.843^**^	1

Note: *P < 0.05,**P < 0.01.

Table 4 presents the direct, indirect, and total effects of the various latent variables. HL exerted a negative influence on FOP (β = -0.300, 95%CI[-0.380,-0.211]) and a positive influence on SE and SS (SE; β = 0.434, 95% CI [0.359, 0.505]) and social support (SS; β = 0.356, 95% CI [0.244, 0.451]). SS had a positive effect on SE (β = 0.131, 95% CI [0.030, 0.229]) but no statistically significant effect on FOP (β = 0.004, 95% CI [–0.076, 0.011]). SE had a negative influence on FOP (β =−0.281, 95% CI [–0.386, − 0.161]). The mediation analysis revealed two significant indirect pathways: (1) The HL→SE→FOP pathway showed an indirect effect of -0.122 (β = 0.387 × -0.281), accounting for 40.67% of the total effect (-0.300); (2) The HL→SS→SE pathway demonstrated an indirect effect of 0.047 (β = 0.356 × 0.131), representing 10.83% of the total effect (0.434). These findings indicate that SE serve as a partial mediator in the relationship between HL and FOP. Notably, although SS did not have a direct effect on FOP, it indirectly contributed to reducing fear of progression through its positive impact on SE.

As shown in Fig. 1, the model explains 15.4% of the variance in FOP (R² = 0.154), 20.3% in self-efficacy (R² = 0.203), and 12.7% in social support (R² = 0.127). This suggests that the proposed theoretical framework captures meaningful psychological mechanisms underlying FOP among lung cancer patients.

**Table 4:** Factor effect breakdown of FOP.

Relationship between variables	Direct Effects	Indirect Effects	Total Effects
H1: HL→SS	0.356(0.244 ~ 0.451)	0.000	0.356(0.244 ~ 0.451)
H2: HL→SE	0.387(0.302 ~ 0.466)	0.047(0.016 ~ 0.091)	0.434(0.359 ~ 0.505)
H3: HL→FOP	-0.178(-0.380~-0.211)	-0.122(-0.182~-0.070)	-0.300(-0.380~-0.211)
H4: SS→SE	0.131(0.030 ~ 0.229)	0.000	0.131(0.030 ~ 0.229)
H5: SS→FOP	0.000	-0.037(-0.076~-0.011)	-0.037(-0.076~-0.011)
H5: SE→FOP	-0.281(-0.386~-0.161)	0.000	-0.281(-0.386~-0.161)

## Discussion

This study aimed to better understand the complex factors that influence FOP among lung cancer patients. We investigated the relationship between HL and FOP, and further examined the potential influence and mediating roles of SS and SE in this association. The findings empirically validate a psychosocial model linking health literacy to fear of progression through sequential mediation by self-efficacy and social support utilization.

In this study, the mean FOP score (31.20 ± 9.28) was, slightly higher than that reported in studies conducted in Beijing (30.3 ± 9.48)^6^ and Guangzhou(29.03 ± 9.43)^11^, but lower than those from Henan (35.39 ± 8.03)^28^, Hunan (36.3 ± 9.2)^14^, and Korea (36.5 ± 12.2)^32^. Cross-cancer comparisons likewise reveal marked heterogeneity (17,26), attributable to differences in demographic composition, cultural context, healthcare systems, and sample size (14,27). Although there is a lack of evidence that cancer type is significantly associated with FOP, patients with lung cancer generally experience relatively high levels of FOP, largely due to the high prevalence of psychological distress and the poor prognosis of the disease itself^32,33^. Lung cancer is often accompanied by prominent respiratory symptoms such as dyspnea, cough, and chest pain, and is associated with rapid progression, high recurrence rates, and poor prognosis^33^. This makes them more likely to interpret physical discomfort as signs of disease deterioration, thereby exacerbating psychological burden.

Notably, this study found a significant association between FOP levels and disease severity: patients with stage III–IV disease had significantly higher FOP scores than those at earlier stages (*P* < 0.001), which is consistent with findings in other cancer populations, indicating that perceived risk of disease progression is a key predictor of FOP^34^. Likewise, although the association between a family history of cancer and higher FOP levels did not reach conventional statistical significance (*P* = 0.068), it suggests a potential trend toward elevated fear that may be linked to genetic concerns or shared familial experiences of cancer. These findings underscore the necessity for systematic FOP screening and longitudinal dynamic monitoring during routine follow-up, with enhanced vigilance for patients with advanced disease, positive family history, or high symptom burden, facilitating early psychological intervention and quality-of-life preservation.

Pearson correlation analysis revealed a significant negative association between HL and FOP (*r* = -0.298, *p* < 0.01), and SEM confirmed a significant direct negative predictive effect of HL on FOP (β = -0.178). These findings indicate that higher levels of HL are associated with lower levels of FOP, consistent with prior researches^11,35^. This relationship may be explained by the fact that patients with higher HL demonstrate greater proficiency in accessing, understanding, and evaluating health-related information. As a result, they are better equipped to accurately understand disease trajectories and treatment regimens, thereby reducing uncertainty and its associated anxiety^36^. In contrast, individuals with low HL, often hindered by shame or communication barriers, are less likely to proactively ask healthcare providers questions, thus missing opportunities to clarify misconceptions and receive psychological support, which may further intensify fear and anxiety^37^. Moreover, all dimensions of HL, including IAA, CIA, HIW and ESW, were significantly negatively correlated with FOP (all *p* < 0.01), further underscoring the importance of comprehensive HL improvement in alleviating psychological burden. Therefore, meeting patients’ informational needs is not merely a process of knowledge transfer, but a critical intervention for reducing anxiety and enhancing disease understanding^36^. In clinical practice, greater emphasis should be placed on the quality of patient-provider communication, fostering trustful relationships, encouraging patients to express concerns, and enhancing their capacity to participate in shared decision-making.

In addition to its direct protective effect, HL not only exerted a significant direct effect on FOP, but also demonstrated a significant indirect effect through SE (β = -0.122), accounting for 40.67% of the total effect. Path analysis in the present study revealed that HL significantly and positively predicted SE (β = 0.387), indicating that patients with stronger abilities in understanding and applying health information are more likely to develop a “I can cope” mindset^13^. Furthermore, SE exhibited robust psychological regulatory function in this study, showing a significant negative correlation with FOP (*r* = -0.292, *p* < 0.01) and a significant direct negative effect in the structural model (β = -0.281). This suggests that individuals’ confidence in their ability to manage their illness is closely linked to lower levels of FOP, potentially reflecting a greater tendency to adopt adaptive coping strategies, maintain treatment adherence, and preserve emotional stability^15,38^. Patients with high SE are more inclined to proactively express emotional distress and actively participate in shared decision-making with healthcare providers, thereby effectively alleviating psychological burden^39^.

Although bivariate correlation analysis showed a negative correlation between SS and FOP (*r* = -0.118, *P* < 0.01), consistent with previous findings that higher levels of SS are linked to lower FOP^6,40^, interestingly, among the three sub-dimensions of SS, only US, not OS or Subs, was significantly associated with lower FOP. This suggests that even if an individual has an extensive social network (OS) and strong feelings of being cared for (Subs), these supports may not be effective if the patients are unwilling or unable to mobilize that assistance. This also indicates that effective utilization is key to translating support into reduced FOP. This pattern may reflect both the nature of FOP as a future-oriented anxiety that responds more strongly to active coping behaviors than to passive resources, and cultural norms in our sample context, where patients may hesitate to express distress or request help to avoid burdening family members. Therefore, the intervention measures should prioritize not only increasing support availability of SS, but also actively fostering patients’ skills and motivation to utilize it. The hypothesized pathway HL → SS → FOP was not supported, the structural model revealed no significant direct effect. This finding may reflect the double-edged nature of SS: when patients perceive the support they receive as exceeding their capacity to reciprocate, it may trigger feelings of guilt, shame, or burden, particularly the fear of becoming a strain on family members, which in turn may intensify FOP^41^. Moreover, prior research indicates that only SS, perceived as appropriate and effective, can enhance adaptive coping and hope in cancer patients^17,42^.

This finding does not diminish the importance of social support, rather, it highlights its critical indirect contribution to psychological adaptation through the significant chain mediation SS → SE → FoP. This pathway suggests that SS contributes to enhanced SE, which in turn strengthens patients’ capacity to cope with illness-related uncertainty, ultimately mitigating FOP^43^. The result aligns with the core premise of the Buffering Model that SS does not necessarily eliminate FOP directly, but rather exerts its protective effects by bolstering SE, thereby modulating cognitive appraisals and emotional responses to FOP^41^.

Additionally, the present study found a significant positive association between HL and SS (β = 0.356), indicating that patients with greater HL are more likely to recognize, access, and effectively utilize available social resources^44^. This underscores the importance of improving HL as a means to facilitate the effective mobilization and utilization of SS, thereby enhancing its psychological benefits.

## Limitations

This study offers two principal strengths: (i) it simultaneously examines the associations between multiple psychosocial factors including HL, SE, and SS and FOP in lung cancer patients, and (ii) it is the first time to specify the inter-relationships among these variables, thereby providing an evidence-based framework for intervention. Several limitations should, nevertheless, be acknowledged. First, the cross-sectional design makes it impossible to conduct causal inferences, and obscures temporal dynamics. Longitudinal studies are required to explore the trajectory of FOP over time, or qualitative research can be conducted to gain a deeper understanding of their actual experiences with FOP. Second, convenience sampling from two tertiary hospitals in one region limits representativeness; random, community-based recruitment with larger, multi-Centre samples is needed to enhance external validity. Third, several potential risk variables, such as anxiety and illness perceptions, were not investigated, which should be considered in future investigations.

## Conclusions

This study revealed lung cancer patients that have a moderate level of FOP, which was significantly associated with advanced stage. Furthermore, the study highlights the complex relationships among several factors related to FOP in lung cancer patients. HL showed the strongest association with FOP, demonstrating both a significant direct link and an indirect association through its positive relationship with SE, underscoring its potential centrality in psychological adaptation. Among the dimensions of SS, US is of greater clinical significance than OS or Subs, indicating that patients’ skills to actively engage with available support may be more closely tied to lower FOP than the mere presence or perception of support. The chain mediation analysis further revealed a potential indirect pathway whereby higher levels of SS, particularly US, were associated with greater SE, which in turn was linked to lower FOP, pointing to a cumulative psychosocial process. These findings suggest that comprehensive interventions should concurrently target HL, SE, and support utilization. Rather than simply expanding social networks, efforts could focus on enhancing patients’ skills and confidence in accessing and using support, alongside promoting adaptive coping strategies. Integrating health education, psychological screening, and practical skills training into routine cancer care may be particularly beneficial for high-risk individuals, such as those with a prolonged disease duration of cancer, to enable more tailored psychosocial support. Healthcare providers should monitor HL levels and routinely screen for mental comorbidities. Future studies are needed to test whether interventions grounded in this integrated model improve long-term psychological outcomes in lung cancer survivors.

## Data Availability

The datasets used and/or analyzed during the current study are available from the corresponding author on reasonable request.

## Abbreviations

IARC: International Agency for Research on Cancer
FOP: Fear of disease progression
SE: Self-efficacy
HL: Health literacy
SS: Social support
SEM: Structural equation model
FOP-Q-SF: Fear of Progression Questionnaire-Short Form
PH: Physical health
SF: Social family
HeLMS: Health Literacy Management Scale
IAA: Information acquisition ability
CIA: Communication interaction ability
HIW: Health improvement willingness
ESW: Economic support willingness
SSRS: Social Support Rating Scale
OS: Objective support
Subs: Subjective support
US: Utilization of support
SUPPH: Strategies used by people to promote health
PA: Positive attitude
SD: Self-decision
SDP: Self-decompression
CFI: Comparative fit index
GFI: Goodness-of-fit index
NFI: Normed fit index
RMSEA: Root mean square error of approximation
